# Antioxidative, Metabolic and Vascular Medicinal Potentials of Natural Products in the Non-Edible Wastes of Fruits Belonging to the *Citrus* and *Prunus* Genera: A Review

**DOI:** 10.3390/plants13020191

**Published:** 2024-01-10

**Authors:** Chika I. Chukwuma

**Affiliations:** Centre for Quality of Health and Living (CQHL), Faculty of Health and Environmental Sciences, Central University of Technology, Private Bag X20539, Bloemfontein 9300, Free State, South Africa; chykochi@yahoo.com or cchukwuma@cut.ac.za; Tel.: +27-(0)-51-507-4028

**Keywords:** *Citrus*, *Prunus*, fruit waste, metabolic diseases, oxidative stress, phytochemistry

## Abstract

Diabetes mellitus and related metabolic and vascular impairments are notable health problems. Fruits and vegetables contain phenolics that are beneficial to metabolic and oxidative health and useful in preventing associated disease. Scientific evidence has shown that some bioactive phenolics are more abundant in the non-edible parts (especially the peels) of many fruits than in their respective edible tissues. Fruits belonging to the *Citrus* and *Prunus* genera are commonly consumed worldwide, including in South Africa, and their non-edible wastes (peel and seed) have been shown to have antioxidative, metabolic and vascular pharmacological potentials and medicinal phytochemistry. It is therefore imperative to evaluate the pharmacological actions and phytochemical properties of the non-edible wastes of these fruits and understand how they could potentially be of medicinal relevance in oxidative, metabolic and vascular diseases, including diabetes, oxidative stress, obesity, hypertension and related cardiovascular impairments. In the absence of a previous review that has concomitantly presented the medicinal potentials of fruits wastes from both genera, this review presents a critical analysis of previous and recent perspectives on the medicinal potential of the non-edible wastes from the selected *Citrus* and *Prunus* fruits in metabolic, vascular and oxidative health. This review further exposes the medicinal phytochemistry, while elucidating the underlying mechanisms through the fruit wastes potentiates their therapeutic effects. A literature search was carried out on “PubMed” to identify peer-reviewed published (mostly 2015 and beyond) studies reporting the antidiabetic, antioxidative, antihypertensive, anti-hyperlipidemic and anti-inflammatory properties of the non-edible parts of the selected fruits. The data of the selected studies were analyzed to understand the bioactive mechanisms, bioactive principles and toxicological profiles. The wastes (seed and peel) of the selected fruits had antioxidant, anti-obesogenic, antihypertensive, anti-inflammatory, antidiabetic and tissue protective potentials. Some phenolic acids and terpenes, as well as flavonoids and glycosides such as narirutin, nobiletin, hesperidin, naringin, naringenin, quercetin, rutin, diosmin, etc., were the possible bioactive principles. The peel and seed of the selected fruits belonging to the *Citrus* and *Prunus* genera are potential sources of bioactive compounds that could be of medicinal relevance for improving oxidative, metabolic and vascular health. However, there is a need for appropriate toxicological studies.

## 1. Introduction

Diabetes is a chronic endocrine disorder that adversely alters the metabolism of carbohydrates, proteins, fat, electrolytes and water, predominantly due to a derangement in insulin production and/or action [1]. It is characterized by hyperglycemia, which is majorly due to the loss of insulin secretion and/or action in target tissues. Type 2 affects 90–95% of people with diabetes [2]. It is caused by progressive insulin resistance [3]. In peripheral tissues, this results in impaired circulating glucose uptake and compromised nutrient metabolism, which progressively compromises the function of pancreatic beta cells [3]. Insulin resistance can also lead to dyslipidemia, elevated inflammatory markers and the production of reactive oxygen species (ROS), which can deplete vascular endothelial nitric oxide, leading to elevated blood pressure (BP) [2,4,5].

Elevated blood pressure (systolic/diastolic blood pressures of ≥140/90 mmHg) is the main clinical sign of hypertension [4]. There are several metabolic processes that can influence the development of hypertension, notably, the renin–angiotensin–aldosterone system, if not properly regulated [4]. The catalytic action of renin and angiotensin converting enzyme (ACE) produces angiotensin II, a peptide that is key in mediating most pathological effects relating to hypertension, including vascular smooth muscle constriction, nitric oxide (a key vasodilator in the vasculature) depletion and blood pressure elevation [4].

Obesity, on the other hand, remains one of the main risk factors of type 2 diabetes because it predisposes people to insulin resistance and metabolic syndrome [3]. Through various mechanisms, adiposity and fat accumulation promote insulin resistance [3]. An increase in free fatty acid release and adipokine deregulation are some mechanistic drivers of pro-inflammatory responses causing obesity-related insulin resistance and type 2 diabetes [3].

Persistent hyperglycemia in type 2 diabetes can lead to oxidative damage of the vessels, which may be microvascular (retinopathy, neuropathy and nephropathy) and macrovascular (cardiovascular impairments) [2,4,5]. Thus, diabetes co-exists with several metabolic and vascular impairments. Oxidative stress has been implicated as a major mediator and culprit in the development and progression of diabetic complications [5]. Elevated blood glucose (BG) can lead to increased glycation process, lipid peroxidation (LPO) and production of pro-oxidants, which can oxidatively damage vital tissues and organs, trigger pro-inflammatory responses and lead to diabetic complications [5]. ROS, such as hydrogen peroxide, can cause a marked decrease in insulin signaling and glucose transport in target cells [5]. Stress-activated serine kinases are, also, involved in the pathogenesis of oxidant-induced insulin resistance, and excess mitochondrial superoxide ion and hydrogen peroxide production plays a pivotal role in causing insulin resistance in the skeletal muscle [5].

The available information suggests that diabetes is emerging as a significant health problem in Africa, including in South Africa [2,6]. Other than direct health care expenditure, premature deaths and disabilities are some of the diabetes-related detrimental outcomes that pose a serious burden on the quality of life of patients and their families. In South Africa, the most affected has been the low–middle-income population [7], who struggle to afford quality healthcare. Moreover, it has been reported that therapeutic interventions, including exercising, dietary adjustments and the use of medication, are more prevalent among patients with a higher socio-economic status as compared to the lower counterparts, perhaps due to affordability and accessibility [8]. Presumably due to affordability and availability, most of them rely on fruits, vegetables and indigenous plant concoctions as complementary and/or alternative approaches to preventing and managing several chronic non-communicable diseases, including diabetes and vascular complications [9].

Fruits contain natural antioxidants, which can ameliorate oxidative stress and impede the progression of related metabolic and cardiovascular diabetic complications [10]. Advances in research have shown that the non-edible wastes, such as the peels of many fruits, contain more phenolics and other phytochemicals than their respective edible tissues [11,12,13], which could be of medicinal relevance for chronic diseases, including diabetes, hypertension, obesity and cardiovascular complications. This may be useful for a country like South Africa, where non-communicable diseases, including metabolic and vascular diseases, are increasing and comprehensive healthcare is unaffordable to many.

It has been documented that fruits including *Citrus* fruits (orange, grapefruit, lemon, lime and nartjie), *Prunus* fruits (peach, cherry, plum, apricot, etc.) are commonly consumed in South Africa and thus generate non-edible wastes [14] that could be explored as potential natural sources of bioactive principle for the management of oxidative stress, diabetes and related impairments. It is, therefore, worthy to evaluate the pharmacological and phytochemical properties of the non-edible (peel and seed) wastes of these fruits and understand how they could potentially be of medicinal relevance in oxidative, metabolic and vascular diseases, which include diabetes, oxidative stress, obesity, hypertension and related cardiovascular impairments. Moreover, documented evidence has shown the health benefits of plant-derived polyphenols on metabolic, vascular and oxidative health [15,16,17].

To the best of our knowledge, there is presently no review that has concomitantly documented the antioxidative, metabolic and vascular medicinal potentials of the non-edible wastes of *Citrus* and *Prunus* fruits. This review was, therefore, aimed at reviewing the antidiabetic, antilipidemic, antihypertensive, antioxidative, anti-inflammatory and cardio/hepatoprotective pharmacological findings on the non-edible parts of selected fruits belonging to the *Citrus* and *Prunus* genera that have been reported as notable contributors to fruit wastes in South Africa [14]. This may provide an evidence-based platform to promote the medicinal utilization of South African fruit wastes.

## 2. Review Strategy

The scientific and common names of the selected fruits were confirmed on “The Plant List” database (http://www.theplantlist.org (accessed on 23 October 2022)). A literature search was carried out on “PubMed”. The aim was to identify peer-reviewed published (mostly studies from 2015 and beyond) data reporting the antidiabetic, antilipidemic, antihypertensive, antioxidative, anti-inflammatory and cardio/hepatoprotective effects of the non-edible parts of the selected fruits. The keywords included a combination of the name (common or scientific name) of fruit, its non-edible part and the relevant bioactivity. All the search outcomes were carefully looked at to select only the studies that were relevant to the scope of this review. The selected studies were analyzed to understand the bioactive mechanisms, bioactive principles and toxicological profiles of the non-edible fruit part.

## 3. Ethnomedicinal, Pharmacological and Phytochemical Properties of the Non-Edible Fruit Parts Selected Fruits

Discussed below are the ethnomedicinal profiles and phytochemistry, as well as the in vitro, in vivo and clinical antidiabetic, antilipidemic, antihypertensive, antioxidative, anti-inflammatory and cardio/hepatoprotective effects of the non-edible parts of the selected fruits.

### 3.1. The Citrus Fruits

**Figure 1:**
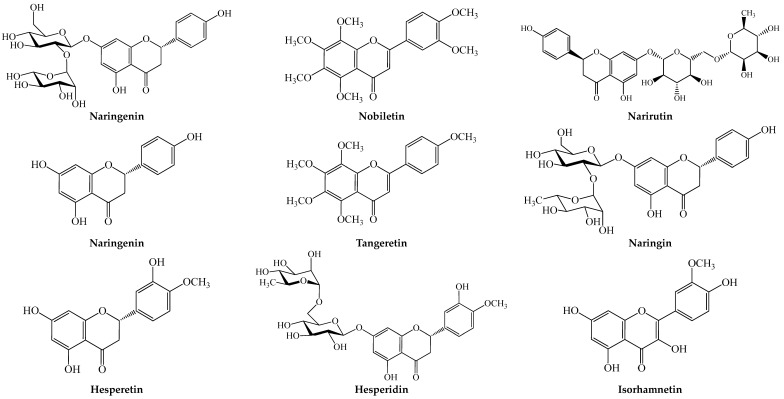

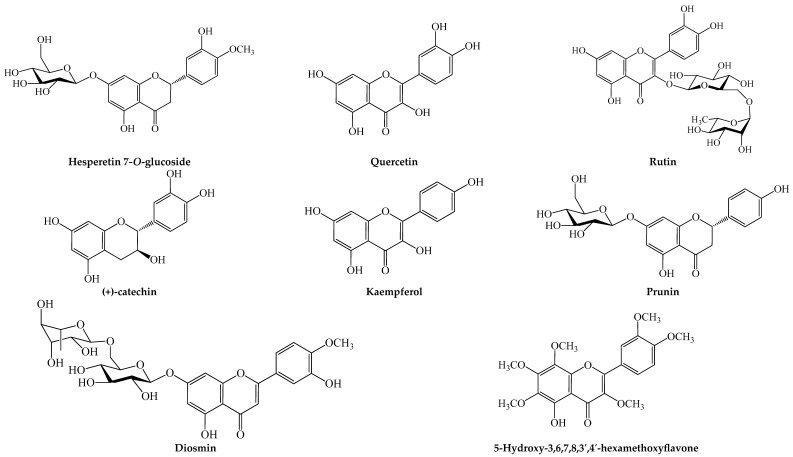
Structures of flavonoids and related glycosides identified in peel and seeds of the selected *Citrus* fruits.

**Figure 2:**
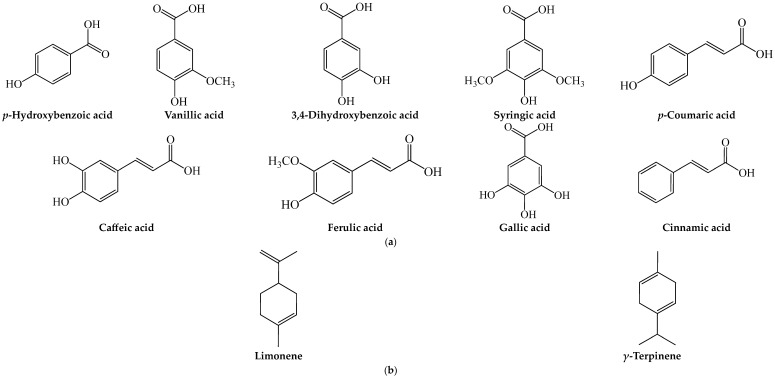
Structures of (**a**) phenolic acids and (**b**) terpenes identified in or isolated from peel and seeds of the selected *Citrus* fruits.

The *Citrus* fruits belong to the Rutaceae family and *Citrus* genus. They include oranges, grapefruits, nartjies, lemons and limes and reportedly generate the highest fruit wastes in South Africa [14]. The seeds and peels are the main non-edible wastes from the *Citrus* fruits. About 79% of the wastes from the citrus fruits emanate from the non-edible parts that are discarded after consumption [14]. Pharmacological studies have given credence to the antidiabetic, antioxidative and anti-inflammatory potential of the peel and seeds of *Citrus* fruits, which is largely influenced by the presence several phenolic acids, terpenes and flavonoids, including narirutin, narigenin, nobiletin, tangeretin, rutin, hesperetin, hesperidin and methoxyflavones [18,19,20,21] (Figure 1 and Figure 2). Table 1 summarizes the oxidative and metabolic pharmacological properties of the non-edible parts of the *Citrus* fruits.

The protective effect of the peel has also been shown in vivo. Four weeks of oral treatment (50 mg/kg) with 30% methanol extract in male Wistar rats ameliorated N-acetyl-p-aminophenol-induced liver damage by reducing serum ALT, AST, ALP, inflammatory maker (TNF-α and IL-4), hepatic LPO and pro-apoptotic activity and improving hepatic antioxidant status and histopathological deteriorations [29]. The effect of the extract was comparable to that of naringin (20 mg/kg bw) and naringenin (20 mg/kg bw), which were two of the bioactive principles (diosmin, gallic acid, naringin, rutin, hesperidin, quercetin, naringenin and hesperetin) identified with HPLC-MS [29] (Figure 1).

#### 3.1.1. Orange (*Citrus* × *sinensis* (L.) Osbeck)

Oranges are popularly consumed in South Africa due to their sweet and pleasant taste. The peel and seeds are the major non-edible wastes of the fruit. The peel is traditionally used to treat symptoms of indigestion and respiratory tract inflammation [22]. Pharmacological evidence shows the potential of its peel and seeds to potentiate glycemic control, ameliorate diabetes and obesity, reduce tissue inflammation and protect against tissue oxidative damage.

The peel extracts and fractions have been shown to scavenge reactive radicals and reduce Fe^3+^ in vitro, suggesting its antioxidant potential [18,21]. The boiling water extract of the peel suppressed lipopolysaccharide (LPS)- and IFN-γ-induced NO production in RAW 264.7 cells and reduced the expression of iNOS and COX-2 in the cells [18]. According to Chen et al. [18], the anti-inflammatory action of the boiling water peel extract was dose-dependent (1–4 mg/mL) and stronger than a mixture of flavonoids (narirutin, hesperidin, nobiletin and tangeretin) at equivalent concentrations. In HepG2 cells, it suppressed t-BPH-induced oxidative stress and cytotoxicity by increasing the antioxidant status and suppressing ROS production, LPO, caspase-3 activation and pro-apoptotic signaling, which suggests that it has a protective effect against oxidative stress and inflammation-induced tissue damage and apoptosis [27]. HPLC analysis suggests narirutin, hesperidin, hesperetin, nobiletin and tangeretin as the potent bioactive principles [18,27] (Figure 1).

Extracts and phytochemicals from the peel have also been shown to demonstrate antidiabetic and anti-obesogenic effects. In 3T3-L1 adipocytes, 5-Hydroxy-3,6,7,8,3′,4′-Hexamethoxyflavone (Figure 1) isolated from the peel inhibited adipogenesis and fat/lipid accumulation in 3T3-L1 adipocytes by downregulating PPARγ, C/EBPs, FAS and ACC and upregulating AMPK [23]. The ethanol and methanol extracts of the peel demonstrated in vivo antidiabetic effects by reducing BG, improving the lipid profile, modulating insulin signaling markers, glucose metabolism and glycogen synthesis, improving pancreatic histology, antioxidant status and insulin secretion and protecting against diabetic nephropathy [24,25,26].

Extracts, essential oil and protein isolates from the seed were reported to exhibit in vitro 2,2-diphenyl-1-picrylhydrazyl (DPPH) radical scavenging and antiglycation activities [30,31,32,33]. The protein isolates, however, further exhibited an in vitro inhibitory effect on α-glucosidase, α-amylase and ACE [33], which suggests that the seeds may be a source of bioactive proteins for postprandial glycemic control and management of hypertension. Phytochemicals identified in the methanol extracts of the seeds included mostly phenolic acids (gallic acid, 3,4-dihydroxybenzoic acid, syringic acid, *p*-coumaric acid, caffeic acid, ferulic acid and cinnamic acid) and flavonoids ((+)-catechin, rutin, quercetin, kaempferol, naringenin and isorhamnetin) (Figure 1 and Figure 2a) which potentiated its radical scavenging effect [33].

In a toxicological context, extracts and/or isolates of the peel appear not to be toxic at effective doses. While methoxyflavone isolated from the peel demonstrated anti-adipogenic effects in 3T3-L1 adipocytes at concentrations ranging from 5 to 20 µM, up to 100 µM of the methoxyflavone did cause notable toxic effects to the cells [23]. In RAW 264.7 cells, boiling water extract of the peel (1–4 mg/mL) and some constituent flavonoids (narirutin, nobiletin and tangeretin) at 2.5–80 µg/mL exerted anti-inflammatory actions by inhibiting NO production [18]. Although the cytotoxicity data of the extract were missing, nobiletin, which was the most effective among the flavonoids, did not adversely affect the cells’ viability. However, in HepG2 cells, the antioxidant action of boiling water extract (50–500 µg/mL) was accompanied by anti-apoptotic and anti-cytotoxic effects [27], suggesting safety at effective doses. In rats, the peel’s methanol extract (50 and 100 mg/kg bw for 30 days; p.o.) exacted antidiabetic effects, while up to 1000 mg/kg bw (p.o.) of the extract was not toxic or caused adverse metabolic alterations 14 days post-treatment in the rats. On the other hand, the reviewed studies lacked toxicological data on the seeds.

#### 3.1.2. Naartjie (*Citrus unshiu* (Yu.Tanaka ex Swingle) Marcow)

The common non-edible wastes from this fruit are the peel and seeds. However, recent antidiabetic and antioxidative studies have mostly been limited to the peel. Traditionally, the peel is used for treating cough, cold, asthma, nausea, digestive problems and inflammation of the skin and respiratory tract, as well as improving blood circulation [19,34,35,36]. Pharmacological studies suggest the antidiabetic, anti-obesogenic, antihypertensive, antioxidative, anti-inflammatory and tissue protective effects of the peel.

The subcritical water extract and 70% ethanol flavonoid-rich extract of the peel possess in vitro radical scavenging and Fe^3+^ reducing antioxidant activities [20,39]. The peel extracts also demonstrated in vitro inhibition on ACE, pancreatic lipase, α-glucosidase and xanthine oxidase [20,39], suggesting its potential to control hyperglycemia, detrimental weight gain, hypertension and ROS production. The major HPLC-identified flavonoids in peel extracts were narirutin, narigenin, prunin, nobiletin, tangeretin, hesperetin-7-*O*-glucoside, hesperetin and hesperidin (Figure 1), which could influence the bioactivates of the extracts.

In different cell types, extracts of the peel exerted protective effects. Fermented water and ethanol peel extracts suppressed LPS-induced inflammation in RAW 264.7 macrophages by suppressing NO production, iNOS and COX-2 expression and the secretion of inflammatory cytokines (IL-6, TNF-α and PGE_2_) [38]. Furthermore, the boiling water extract of the peel suppressed H_2_O_2_-induced oxidative damage in neuronal cells (HT22 cells) by reducing cytotoxicity or cell death and the expression of pro-apoptotic signaling proteins (p-JNK, p-p38, caspase 3 and Bcl-2) [37]. The anti-cytotoxic and anti-apoptotic effect of the extract was comparable to that of nobiletin [37].

In vivo, the peel water extract acutely (20 mg/kg bw for 5 h; p.o.) ameliorated aspirin-induced stomach ulceration and bleeding and was as potent as hesperidin [36]. Also, the ethyl acetate fraction of the peel’s 70% ethanol extract improved glycemic control and the serum lipid profile, reduced hepatic lipid accumulation and oxidative stress, serum ALT and AST, upregulated the expression of hepatic Nrf-2, NQO1 and fatty acid oxidation genes and downregulated the expression of hepatic inflammatory factors (IL-1β, IL-6, MCP-1 and TNF-α) in high-fat diet (HFD)-fed male C57BL/6J mice following 10 weeks of feeding a diet containing 0.2 and 0.5% of the fraction [19]. The antidiabetic, antilipidemic and anti-inflammatory effects of the extract were comparable to those of a diet containing 0.5% resveratrol [19]. The potent effect of the fraction may be attributed to nobiletin and the methoxyflavones present in the fraction [19], which has been documented previously [54,55]. In obese patients, 4 weeks of consumption of peel pellets (18 mg per day) exerted anti-obesogenic effects by reducing body weight, body mass index (BMI), waist circumference, total and LDL cholesterol and triglyceride (TG) [35]. HPLC identified narirutin, nobiletin and hesperidin (Figure 1) in the pellets.

From a toxicological perspective, the antioxidant effect of the peel’s boiling water extract in neuronal cells (HT22 cells) was accompanied by anti-apoptotic and anti-cytotoxic effects [37], suggesting that it may be safe for medicinal purposes following more appropriate in vivo toxicological evaluation. Similarly, water extracts of the fermented peel showed anti-inflammatory effects in RAW 264.7 macrophages but were non-toxic to the RAW 264.7 cells and HaCaT cell line even at the effective doses (up to 100 µg/mL).

#### 3.1.3. Grapefruit (*Citrus* × *paradisi* Macfad)

The peel and seeds are the non-edible wastes of grapefruits. A recent moderate increase in the consumption of grapefruit has also increased the wastes from the fruit. In the north-eastern part of Africa, the peel is used to treat catarrh and malaria [40]. The seeds are, however, used in the south-western part of Nigeria to manage diabetes and obesity [44].

A flavonoid-rich extract (70% ethanol) of the peel was shown to exhibit an in vitro radical scavenging and cellular (HepG2) antioxidant effect, as well as an in vitro pancreatic lipase inhibitory effect [39], suggesting that it may mitigate cellular oxidative damage and the development of obesity. Hesperidin and hesperetin (Figure 1) were identified as possible bioactive flavonoids [39]. Ex vivo and in situ studies showed that the 60% ethanol extract of the peel exhibited a coronary vasodilation effect and suppressed vascular resistance [41], suggesting potential relevance in managing hypertension and cardiovascular problems. Studies in male Wistar rats showed that a 3-day pre-treatment with the narirutin-rich fraction of the fruit peel dose-dependently protected against isoproterenol-induced myocardial injury, with the highest dose (200 mg/kg bw) being as effective as a 5 mg/kg bw Atenolol pre-treatment [43]. In STZ-induced diabetic Wistar rats, a 12-day treatment (400 mg/kg bw, p.o.) with the peel’s ethanol extract improved wound healing by promoting tissue growth and collagen synthesis [28]. Additionally, the peel powder ameliorated trinitrobenzenesulfonic acid-induced colonic injury, inflammation and oxidative stress in Wistar rats following 15 days of feeding a diet containing 8% of peel powder [42].

The extracts of the seeds have also been shown to demonstrate in vivo glycemic control and antilipidemic, antioxidative and hepatic tissue protective properties. A thirty-day treatment (100, 300 and 600 mg/kg bw, p.o.) with the seed’s methanol extract dose-dependently reduced fasting BG (FBG), BMI, atherogenic index (AI), coronary risk index (CRI), LDL cholesterol and total cholesterol and increased HDL cholesterol in Wistar rats [44]. In situ, a commercial grapefruit seed extract (Herb-Pharma s.r.o., Velke Ludnice, Slovakia) ameliorated acute hemorrhagic pancreatitis in male Wistar rats [45]. Additionally, a 7-day pre-treatment (200, 400 and 600 mg/kg bw) with the peel’s extract (70% ethanol) dose-dependently reduced the paracetamol-induced serum elevation of ALT, AST and ALP, suggesting a hepatoprotective effect [46].

Toxicological experiments suggest that the hydroalcoholic extract of grapefruit seeds may be safe within the effective dose (600 mg/kg bw) in the context of its protective effect against paracetamol-induced tissue or hematological adverse alterations in mice [46]. In mice, both oral and i.p. administration of the extract (up to 3800 mg/kg bw) was non-toxic or non-lethal for up to 3 days post-administration [46].

#### 3.1.4. Lemon (*Citrus limon* (L.) Osbeck) and Key Lime (*Citrus* × *aurantiifolia* (Christm.) Swingle)

The peels of lemons and limes are popularly used for culinary purposes. In West Africa, lemon and lime peels are combined with other herbal ingredients and traditionally used to treat hypertension [47]. Essential oil from lime peel is used for treating colds, sore throat, bronchitis, asthma and arthritis, as well as to manage obesity [50].

The methanol and ethanol extracts, as well as essential oils, of the peels of lemons and limes have been shown to exhibit radical scavenging and Fe^3+^ reducing antioxidant activity [32,39,48,51]. The methanol extract of lime peel showed the presence of mostly phenolic acids (gallic acid, 3,4-dihydroxybenzoic acid, syringic acid, *p*-coumaric acid, caffeic acid, ferulic acid and cinnamic acid) and flavonoids ((+)-catechin, rutin, quercetin, kaempferol, naringenin and isorhamnetin) (Figure 1 and Figure 2a), which influenced its potent radical scavenging activity [32]. The flavonoid-rich (hesperidin and hesperetin) ethanol extracts of lemon peel also exhibited an antioxidant effect in hepatocytes [39] and a proliferative effect in isolated mouse splenocytes [48], suggesting its potential to exert immunostimulatory effects and protect against cellular oxidative damage. In diabetic Wistar rats, the ethanol extract of lemon peel (400 mg/kg bw, p.o.) improved wound healing by promoting tissue growth and collagen synthesis [28].

Furthermore, the antihypertensive, anti-obesogenic and anti-coronary effects of the peels of lemon and lime have been demonstrated as in vitro pancreatic lipase, ACE and phosphodiesterase type 5 (PDE-5) inhibitory action [39,47]. According to Ademosun et al. [47], the in vitro inhibitory action of lemon and lime peels on ACE and PDE-5 was demonstrated by the acidified methanol (80:20% *v*/*v* of methanol and 1 N HCl solution, respectively) extracts. From a chemical point of view, this concentration of HCl used for extracting phenolics may be adopted with caution. This is because using high concentrations of HCl for extraction could cause complete ionization of all phenolics, making them highly vulnerable to oxidation and/or chemical modification.

In vivo, sub-chronic to chronic administration of lime peel powder or essential oil to hypercholesterolemic or HFD-fed animals ameliorated hyperlipidemia and hypercholesterolemic and reduced AI as well as fatty streaks in the coronary arteries and aorta [51,53]. In fact, cholesterol and TG were reduced to a normal or near-normal range, while the AI was reduced by 86%, suggesting the possible benefits of lime peel on vascular health. GC-MS analysis showed that the essential oil contained bioactive terpenes, limonene and γ-terpinene [51] (Figure 2b), which have been shown to suppress dyslipidemia and hyperlipidemia in animal models [56,57]. Supporting clinical data showed that a 4-week oral treatment with lime peel powder reduced BMI, systolic and diastolic BP and total and LDL cholesterol in overweight and obese adolescents [52].

Studies on the seeds of lime also revealed that it possesses antidiabetic, antilipidemic, antioxidative and tissue protective potentials. A twenty-eight day treatment (100, 200 and 400 mg/kg bw, p.o.) with the water extract in STZ-induced diabetic rats reduced BG, HbA1c, intestinal α-glucosidase activity and serum TG, HDL cholesterol and enzyme biomarkers of renal and hepatic injury, increased serum insulin and improved the erythrocyte, renal and hepatic antioxidant status [49]. The BG-lowering and intestinal α-glucosidase inhibitory effect of the extract was stronger and/or comparable to that of 20 mg/kg bw Acarbose [49], suggesting the postprandial glycemic control potential of lime seeds.

In summary, while the seeds of the *Citrus* fruits possess some pharmacological properties, the peels appear to be more studied, with data indicating a host of pharmacological properties. The peels potentiate glycemic control and anti-obesogenic effects by modulating insulin signaling, as well as glucose and lipid metabolism. The peels also offer protective effects to metabolic and vascular tissues by suppressing inflammation, oxidative stress and the apoptotic process. This could be largely attributed to the bioactive phytochemical depositions on the peel, in particular some phenolic acids and terpenes, as well as flavonoids such as naringenin, naringin, narirutin, nobiletin, hesperidin, rutin and the methoxyflavones (Figure 1 and Figure 2).

Toxicological evaluation of the water extract of lemon seeds suggests it may exert antidiabetic and antioxidant effects without posing toxicity concerns. While 28 days of oral administration of 400 mg/kg bw of the extract showed remarkable antidiabetic and antioxidant effects in rats, up to 1000 mg/kg bw oral administration of the extract was not lethal to the rats even after 72 h post-administration [49].

### 3.2. Fruits Belonging to the Prunus Genus

These fruits include the European plum, apricot, sour cherry and peach. They belong to the *Prunus* genus and the family Rosaceae. The seed/kernel of these fruits is the main non-edible waste from the fruit. The phenolic acids and some flavonoids or their derivatives are some of the common phytochemicals in the seeds of these fruits. Table 2 summarizes the reported oxidative and metabolic pharmacological properties of the seed of these fruits from the *Prunus* genus.

#### 3.2.1. Apricot (*Prunus armeniaca* L.)

Traditionally, the seeds of apricots have been used in Asian medicine as an expectorant, antitussive and laxative [58]. Their consumption has also been linked to a reduced risk of several chronic diseases, including metabolic and vascular problems [60]. Pharmacological studies suggest that the seed may have medicinal relevance for diabetes, cardiovascular diseases and oxidative complications.

In vitro, the methanol and water extracts or protein isolates of the seed demonstrated radical scavenging, Fe^3+^ reducing and antiglycation activities [63,64,66]. HPLC showed that the water extract predominantly contains common phenolic acids like caffeic acid, ferulic acid and p-coumaric acid, which may influence its radical scavenging and antiglycation activities [64]. Moreover, the in vitro antiglycation activity of ferulic acid has been documented [81]. The protein isolates of the seed also exhibited in vitro ACE inhibitory and hypocholesterolemic activities [61,66], suggesting the seed may contain bioactive nutrients that may be useful for managing hypertension and atherosclerosis. In both hypercholesterolemic rats and healthy adult women, chronic and sub-chronic administration of the seed flour reduced blood cholesterol and TG [60,65]. The seed flour showed a significant (*p* < 0.05) anti-cholesterolemic effect in the adult women [60] and reduced the AI by 8-fold in hypercholesterolemic rats [65], suggesting the potential medicinal relevance of apricot seeds for cardiovascular problems.

The acute (8 h and 8 days i.p. treatments with 2–4 mg/kg bw) antidiabetic and antioxidative effects of the infrared-assisted detoxified water extract of the seed have been documented in alloxan-induced diabetic Swiss mice [58]. It reduced BG, HbA1c and LPO and increased insulin secretion and catalase activity [58]. The tissue protective effect of the seed has also been demonstrated in rats. Four weeks of feeding a basal diet containing 0.5–1.5 mg/kg bw of seed flour dose-dependently ameliorated dimethylnitrosamine-induced hepatic fibrosis in male SD rats by improving hepatic histology, increasing serum antioxidant enzyme activity and reducing LPO [59]. Also, acute (1 mL p.o.) treatment with seed oil protected against alcohol-induced gastric intestinal injury in Wistar rats by improving epithelial and mucosal histology, improving antioxidant status and reducing inflammation [62].

In a toxicological context, the kernel of apricot has been reported to notably contain a toxic cyanogenic glycoside, amygdaline [58], which raises toxicological concerns. The study reported by Raafat et al. [58] showed that sub-chronic (4 mg/kg bw for 8 days; i.p.) administration of apricot kernel caused a 50.1% mortality rate in the rats, which was attributed to its amygdaline content of 16.1%. However, infrared-assisted detoxification of the kernel reduced the amygdaline content to 1.4% and consequently reduced the mortality to 9.1% at a corresponding dose. Perhaps the toxicity effect may be further reduced by using treatments at 2 or 3 mg/kg bw without compromising the antioxidant and antidiabetic potential, since treatments at these doses (2 or 3 mg/kg bw) also potentiated antioxidant and antidiabetic effects [58].

#### 3.2.2. Sour Cherry (*Prunus cerasus* L.)

Sour cherry is a common ingredient in many South African delicacies, including confections and beverages. The seed is the major non-edible part of the fruit. In South Africa, only about 11% of cultivated plums are processed, while about 89% are consumed or used domestically. This implies that the non-edible seed discarded after fruit consumption contributes to the majority of the waste from this fruit. In some Middle Eastern countries, the seed is used to prepare a syrup and herbal tea infusion for treating fever, liver disease and gonorrhea [67]. Pharmacological studies suggest the potential medicinal relevance of the seed in managing diabetes, cardiovascular ailments and associated inflammatory and oxidative complications.

The water extract of the seed was shown to inhibit in vitro methylglyoxal-induced protein glycation and AGE production [64], while the seed protein isolates exhibited an in vitro antihypertensive effect by inhibiting ACE activity [61]. In HFD-fed rats, chronic to sub-chronic administration of seed flour reduced serum cholesterols and TAG and systolic BP and attenuated HDF-induced adipogenesis by downregulating the expression of adipogenic genes in adipose tissue [68,69], suggesting its potential medicinal relevance for obesity and associated vascular impairments.

The antidiabetic potential as well as the cellular/tissue protective and anti-inflammatory effects of the seed have also been demonstrated. Acute to chronic administration of the flavonoid-rich extracts of the seed in diabetic or hypercholestorolemic animals improved glycemic control and suppressed renal, pancreatic and cardiac damage by reducing retinal thickness, improving pancreatic histology and antioxidant status and reducing cardiac atherosclerotic plaque formation [71,72,74]. In HFD-fed rats, the seed powder exerted neuroprotective effects by downregulating endothelial inflammatory makers (VCAM-1 and ICAM-1) of the frontal cortex and hippocampus [68].

**Figure 3:**
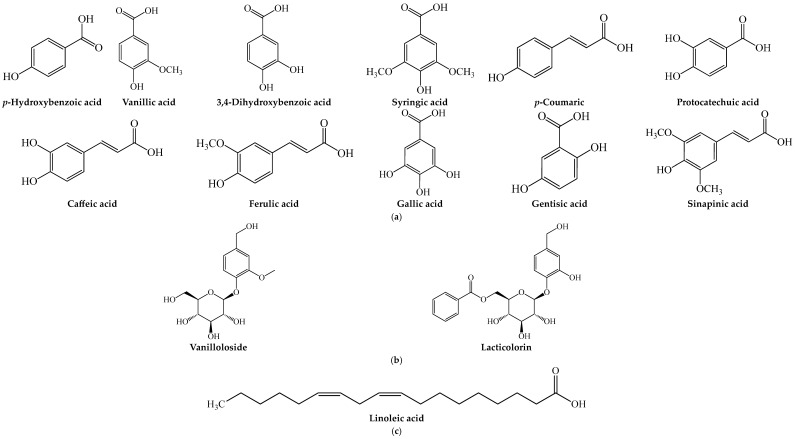
Structures of (**a**) phenolic acids, (**b**) glycosides and (**c**) bioactive fatty acid identified in or isolated from the seeds of the selected *Prunus* fruits.

Furthermore, the hydromethanolic extract of the seed reduced the lipopolysaccharide-induced inflammatory response in leukocytes isolated from diabetic patients by inhibiting or suppressing the upregulation of pro-inflammatory biomarkers (TNF-α and IL-8) and increasing HO-1 expression [73]. In fact, in both obese and cholesterolemic rats, the seed extracts consistently increased HO-1 level in the cells and tissues [71,74], suggesting that the tissue protective effects of sour cherry seed may be linked to immunomodulatory, anti-inflammatory and antioxidant mechanisms. HPLC profiling of the seed’s water extract showed the presence of several antioxidant phenolic acids (*p*-hydroxybenzoic, syringic acid, vanillic acid, *p*-coumaric, caffeic acid, ferulic acid, protocatechuic acid, gallic acid, gentisic acid and sinapinic acid) [64] (Figure 3a), which may influence some of the seed’s bioactivities. In a different study [70], linoleic (Figure 3c) acid was a major fatty acid identified in the seed ethyl acetate extract, which was speculated as a bioactive constituent influencing the anti-inflammatory and tissue protective potential of sour cherry seed. Oral adm. of both the seed extract (200 mg/kg bw) and linoleic acid (20 mg/kg bw) ameliorated HCl- and ethanol-induced gastric lesions in Swiss albino male mice [70]. Concomitantly, they reduced carrageenan-induced nociceptive pain, inflammation and oxidative stress by reducing pro-inflammatory makers (TNF-α and IL-6) and improving the antioxidant status. In fact, their effects were comparable to and/or stronger than the effect of 50 mg/kg bw Ranitidine (an anti-ulcer medication) and 100 mg/kg bw Ibuprofen (an anti-inflammatory medication) [70].

At a cellular and tissue level, protein isolates and extracts of the seed have also been shown to exhibit antioxidant and anti-inflammatory effects. In HeLa cells, the protein isolates or peptides of the fruit’s seed suppressed ROS production and oxidative stress without causing toxicity to the normal cells (HK-2, human renal proximal tubule cells) [75]. The hydromethanolic extract of the seed suppressed tetrachloromethane-induced oxidative stress in isolated rat hepatocytes [78], while compounds (vanilloloside and lacticolorin) (Figure 3b) isolated from the seed methanol extracts suppressed histamine release and the expression of pro-inflammatory cytokines (TNF-α and IL-6) in human mast cells [79]. In SD rats, sub-chronic administration (400 mg/kg bw, p.o.) of the seed’s hydromethanolic extract ameliorated tetrachloromethane-induced hepatic damage by increasing the expression of Nrf2 and NQO1 and improving hepatic histology [78], suggesting the tissue protective effect of the fruit’s seed.

#### 3.2.3. Peach (*Prunus persica* (L.) Batsch 1801)

Peaches are a low-sugar fruit that is widely consumed and used for juice production. The seed is the only non-edible part of the fruit. The seed contributes about 29% of the waste from the fruit in South Africa [14]. In traditional medicine, the seed has been used to treat amenorrhea and rheumatoid arthritis [79].

Pharmacological studies have shown the in vitro antioxidant and antiglycation activities of the water and ethanol extracts of the seed [64,77], which may be influenced by the presence of phenolic acids (*p*-hydroxybenzoic, syringic acid, vanillic acid, *p*-coumaric, caffeic acid, ferulic acid, protocatechuic acid, gallic acid and sinapinic acid) [64] (Figure 3a). Protein isolates from the seed showed in vitro antioxidant, antihypertensive and anti-hypercholesterolemic activities by inhibiting ACE activity and reducing the micellar solubility of cholesterol [61,66,75,76], suggesting the potential use of the seed in the management of cardiovascular impairments.

In vivo, sub-chronic treatment (400 mg/kg bw, p.o.) with the seed’s hydromethanolic extract ameliorated CCl4-induced hepatic damage in SD rats by increasing the expression of Nrf2 and NQO1 and improving hepatic histology [78]. Interestingly, up to 4000 mg/kg bw (p.o.) of the extract was not toxic or caused mortality in rats, suggesting the possible safety of the hydromethanolic extract within the effective dose [78].

#### 3.2.4. European Plum (*Prunus domestica* L.)

The European plum is the most common plum variety consumed in South Africa. Only about 3% of cultivated plums are processed [14], suggesting that a great majority of the plums produced in South Africa are consumed or used domestically. This implies that the non-edible seeds discarded after fruit consumption contribute to the majority of the waste from this fruit. Unfortunately, pharmacological studies on the seeds have been limited to in vitro antioxidant and antihypertensive evaluation.

The methanol extract of the seeds has been shown to have in vitro radical scavenging, Fe^3+^ reducing and xanthine oxidase inhibitory antioxidant activities [80], suggesting it may protect against oxidative stress-mediated pathologies. Phytochemical profiling of the seed’s methanol extract revealed that it contains several antioxidant phenolic acids, including *p*-hydroxybenzoic acid, vanillin, vanillic acid, 3,4-dihydroxybenzoic acid, gallic acid and syringic acid [80] (Figure 3a), which could influence its antioxidant potential. The protein isolates from the seed have been reported to demonstrate in vitro antihypertensive and anti-hypercholesterolemic activities by inhibiting ACE activity and reducing the micellar solubility of cholesterol [61,66], suggesting the potential use of the seed in the management of cardiovascular problems.

In summary, the seeds of the above-mentioned fruits belonging to the *Prunus* genus are rich in several bioactive phenolic acids which may influence their reported activities. The seeds suppressed chemical-, diet- and disease-induced oxidative and inflammatory tissue damages by improving the antioxidant status and immunomodulation response and suppressing inflammatory and apoptotic processes. The seeds also potentiated glycemic control, ameliorated obesity-related lipid alterations and could be a potential source of antihypertensive peptides.

## 4. Conclusions

**Figure 4:**
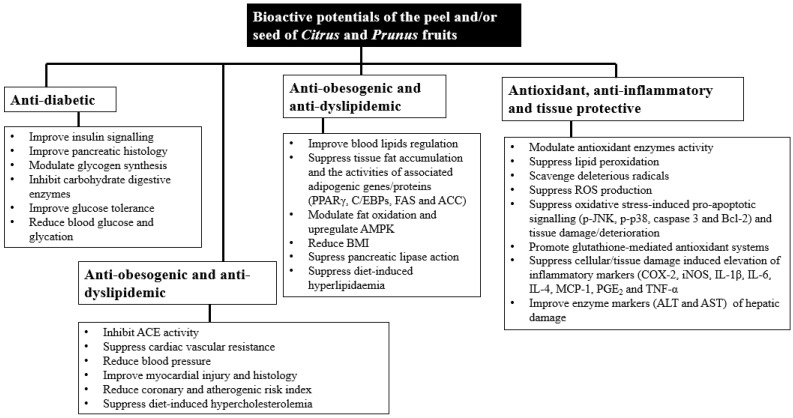
Summary of the bioactive potentials of the peel and/or seeds of *Citrus* and *Prunus* fruits.

Fruits belonging to the *Citrus* and *Prunus* genera are commonly consumed worldwide, including in South Africa. A critical look into the ethnomedicinal and pharmacological data suggests the potential medicinal relevance of the non-edible wastes (peel and seeds) from these fruits in the management of metabolic, vascular and oxidative health. A review of the existing pharmacological evidence showed that the peel and seeds of most citrus fruits have glycemic control, anti-obesogenic, anti-inflammatory, anti-atherogenic, tissue protective and antioxidant potentials (Figure 4).

While some notable phenolic acids may be partly influential in the antioxidant and tissue protective potentials, flavonoids including naringin, naringenin, nobiletin, hesperidin, hesperetin, narirutin, rutin, quercetin and related glycosides, as well as bioactive terpenes (limonene and γ-terpinene) of essential oils, appear to be bioactive principles influencing the metabolic, vascular and oxidative pharmacological potentials of the peel and seeds of *Citrus* fruits. On the other hand, the seed/pit is the major waste emanating from the *Prunus* fruits. Reported phytochemical profiles showed that the seeds of these fruits contain common phenolic acids, which may be very influential in the reported enzyme inhibitory and antioxidant potentials of the seeds. Also, bioactive glycosides (lacticolorin and vanilloloside) with immunomodulatory and anti-inflammatory potentials were isolated from the seed of peaches. The seeds of *Prunus* may also contain bioactive peptides with antihypertensive and anti-atherogenic potentials.

Despite the above-mentioned pharmacological potentials of the non-edible wastes from the *Citrus* and *Prunus* fruits, the data on their toxicological profiles for some of them are lacking and not evident in the reviewed studies. It is therefore recommended that more appropriate toxicological studies be conducted on the non-edible wastes of these fruits to ascertain their safety for medicinal purposes. Perhaps detoxification methods may be adopted in the processing of the non-edible wastes of these fruits without sacrificing the phytochemical and bioactivity profiles. In conclusion, it is safe to speculate that with more appropriate translational and toxicological investigations the peel and seeds of the selected fruits from the *Citrus* and *Prunus* genera may be of medicinal and bio-economic importance in South Africa, particularly in the context of improving metabolic, vascular and oxidative health. They could be medicinally utilized as functional supplements.

## Figures and Tables

**Table 1:** Ethnomedicinal, pharmacological and phytochemical profiles of the seeds and peel of some fruits belonging to the *Citrus* genus.

Fruit Scientific Name (Common Name)	Parts Used	Traditional Use	Pharmacological Action	Material, Extract, Fraction, Isolate or Formulation Used	Phytochemical Profile of Material, Extract, Fraction, Isolate or Formulation Used (Method of Analysis)	Remarks	References
*Citrus × sinensis* (L.) Osbeck (sweet orange)	Peel	Used to treat symptoms of indigestion and respiratory tract inflammation [22].	Inhibited adipogenesis and fat/lipid accumulation in 3T3-L1 adipocytes by downregulating PPARγ, C/EBPs, FAS and ACC and upregulating AMPK.	5-Hydroxy-3,6,7,8,3′,4′-Hexamethoxyflavone isolated from the peel extract	5-Hydroxy-3,6,7,8,3′,4′-hexamethoxyflavone (HPLC)	Up to 100 µM of isolate showed no notable toxicity on the 3T3-L1 cells.	Wang et al., 2016 [23]
			30 days adm. (50 and 100 mg/kg bw, p.o.) of extracts in STZ and HFD-induced diabetic male Wistar rats reduced FBG, serum ALT and AST, improved GT, pancreatic histology and immunohistochemistry, IR and serum lipid profile and modulated the expression of adipose tissue PPARγ, GLUT-4 and InRec.	Methanol extract	Acacetin, apigenin, artepillin C, caffeic acid, (+)-catechin, chlorogenic acid, cinnamic acid, ferulic acid, kaempferide, naringenin, *p*-coumaric acid, protocatechuic acid, rutin, vanillin and vanillic acid (UHPLC-MS/MS)	Up to 1000 mg/kg bw (p.o.) of extract was not toxic or cause adverse metabolic alterations 14 days post-adm.; The effect of extract was comparable to that of a 100 mg/kg bw Metformin adm.	Sathiyabama et al., 2018 [24]
			4 wks adm. (100 and 200 mg/kg bw, p.o.) ameliorated STZ-induced diabetic nephropathy in rats by increasing creatinine clearance, reducing renal hypertrophy and improving renal histology.	Ethanol extract			Parkar and Addepalli, 2014 [25]
			4 wks adm. (100 mg/kg bw, p.o.) in nicotinamide and STZ-induced diabetic rats improved GT, increased serum insulin and C-peptide levels, hepatic glycogen content and glucose-6-phosphatatse activity, adipose tissue InRec, GLUT-4 and adiponectin expression, reduced hepatic glycogen phosphorylase activity and improved serum lipid profile and antioxidant status.	Ethanol extract		The effect of extract was comparable to that of naringin (100 mg/kg bw) and naringenin (100 mg/kg bw).	Ahmed et al., 2017 [26]
			In vitro DPPH and ABTS radical scavenging activity; In vitro Fe^3+^ reducing activity.	Ethyl acetate fraction of 95% ethanol extract	Narirutin, sinensetin, nobiletin, 4′,5,6,7-tetramethoxyflavone and 3,3′,4′,5,6,7-hexamethoxyflavone		Long et al., 2021 [21]
			In vitro DPPH, ABTS and AAPH radical scavenging activity; Suppressed LPS- and IFN-γ-induced NO production in RAW 264.7 cells; Reduced expression of iNOS and COX-2 in RAW 264.7 cells.	Boiling water extract	Narirutin, hesperidin, nobiletin and tangeretin (HPLC)	The extract was more effective than flavonoid mixture at 1–4 mg/mL.	Chen et al., 2017 [18]
			Suppressed *t*-BPH-induced oxidative stress and cytotoxicity in HepG2 cells by increasing antioxidant enzymes and GHS and suppressing ROS production, LPO, caspase-3 activation and pro-apoptotic signaling.	Boiling water extract	Hesperetin, hesperidin, nobiletin and tangeretin (HPLC)	Extract was non-cytotoxic; Extract (50–500 µg/mL) was more effective than identified flavonoids (10 µg/mL).	Chen et al., 2012 [27]
			12-day treatment (400 mg/kg bw, p.o.) in STZ-induced diabetic male Wistar rats reduced BG and improved wound healing by promoting tissue growth and collagen synthesis.	Ethanol extracts	Carotenoids and vitamin C (non-specialized in vitro spectrometric methods)		Ahmad et al., 2013 [28]
			4 wks adm. (50 mg/kg bw, p.o.) of extracts in male Wistar rats ameliorated N-acetyl-p-aminophenol-induced liver damage by reducing serum ALT, AST, ALP, inflammatory maker (TNF-α and IL-4), hepatic LPO and pro-apoptotic activity, increasing hepatic GSH and antioxidant enzyme activity and improving hepatic histopathological deteriorations.	30% ethanol extract	Diosmin, gallic acid, naringin, rutin, hesperidin, quercetin, naringenin and hesperetin (HPLC-MS)	The effect of extract was comparable to that of naringin (20 mg/kg bw) and naringenin (20 mg/kg bw).	Ahmed et al., 2019 [29]
	Seed		In vitro DPPH radical scavenging activity.	Essential oil	Cholestanol and β-sitosterol (GC)		Jorge et al., 2016 [30]
			In vitro inhibitory activity on BSA glycation.	Pressurized hot water extract and water extract obtained from sequential extraction in increasing polarity (i.e., chloroform to distilled water)			Shakthi Deve et al., 2014 [31]
			In vitro DPPH radical scavenging activity.	Methanol extract	Gallic acid, 3,4-dihydroxybenzoic acid, (+)-catechin, 1,2-dihydroxybenzene, syringic acid, caffeic acid, rutin, *p*-coumaric acid, *trans*-ferulic acid, resveratrol, quercetin, kaempferol, apigenin-7-glucoside, isorhamnetin, naringenin and *trans*-cinnamic acid (HPLC-PDA)		Al Juhaimi et al., 2018 [32]
			In vitro DPPH radical scavenging activity; In vitro ACE inhibitory activity; In vitro α-glucosidase and α-amylase inhibitory activity.	Protein isolates			Mazloomi et al., 2020 [33]
*Citrus unshiu* (Yu.Tanaka ex Swingle) Marcow (naartjie)	Peel	Used for treating cough, cold, asthma, nausea, digestive problems and inflammation of the skin and respiratory tract, as well as improving blood circulation [19,34,35,36].	10 wks feeding of diet containing 0.2 and 0.5% fraction in HFD-fed male C57BL/6J mice improved glycemic control and serum lipid profile, reduced hepatic lipid accumulation and oxidative stress, serum ALT and AST, upregulated expression of hepatic Nrf-2, NQO1 and fatty acid oxidation genes and downregulated expression of hepatic inflammatory factors (IL-1β, IL-6, MCP-1 and TNF-α).	Ethyl acetate fraction of 70% ethanol extract	Isosinensetin, sinensetin, 5,7,3′,4′-tetramethoxyflavone, 5,6,7,4′-tetramethoxyflavone, nobiletin, 3,5,6,7,8,3′,4′-heptamethoxyflavone, 5-hydroxy-6,7,8,3′,4′-pentamethoxyflavone and tangeretin. (UHPLC-QTOF-MS)	The effect of the fractions was comparable and/or more potent than that of diet containing 0.1% resveratrol.	Ke et al., 2020 [19]
			4 wks oral adm. (18 mg) in obese patients reduced BW, BMI, waist circumference, total and LDL cholesterol, TG, ALT and AST.	Peel pellets	Narirutin, nobiletin and hesperidin (HPLC)		Kang et al., 2018 [35]
			Suppressed H_2_O_2_-induced oxidative damage in neuronal cells (HT22 cells) by reducing cytotoxicity or cell death and expression of pro-apoptotic signaling proteins (p-JNK, p-p38, caspase 3 and Bcl-2).	Boiling water extract	Nobiletin and hesperidin (HPLC)	The anti-cytotoxic and anti-apoptotic effect of the extract was comparable that of nobiletin.	Cho et al., 2015 [37]
			In vitro DPPH and AAPH; In vitro Fe^3+^ reducing activity; In vitro pancreatic lipase inhibition; In vitro α-glucosidase inhibition; In vitro xanthine oxidase inhibition; In vitro ACE inhibition.	Subcritical water extracts	Narirutin, narigenin, prunin, nobiletin, tangeretin, hesperetin-7-*O*-glucoside, sinensetin, hesperetin and hesperidin (HPLC)	Subcritical water extraction performed at a flow rate of 1.5 mL/min and temperature of 175 °C showed the most potent activity.	Kim and Lim, 2020 [20]
			Suppressed LPS-induced inflammation in RAW 264.7 macrophages by suppressing NO production, iNOS and COX-2 expression and the secretion of inflammatory cytokines (IL-6, TNF-α and PGE_2_).	*Bacillus subtilis*-fermented water and ethanol peel extracts	Narirutin, narigenin, neoponcirin, nobiletin, tangeretin, rutin, hesperetin and hesperidin (UHPLC-QTOF-MS)	Up to 100 µg/mL of extract did not notably decrease the viability of RAW 264.7 macrophages.	Kim et al., 2019 [38]
			In vitro DPPH, ABTS and oxygen radical scavenging activity; In vitro Fe^3+^ reducing activity; In vitro pancreatic lipase inhibition; Antioxidant activity in HepG2 cells.	70% ethanol flavonoid-rich extract	Hesperidin and hesperetin (HPLC)		Huang et al., 2020 [39]
			Exerted proangiogenic effects in HUVECs by promoting cell proliferation, migration and tube formation and upregulated phosphorylation of angiogenic genes (FAK and ERK1/2).	Peel powder	Narirutin and hesperidin (HPLC and LC-MS)		Lee et al., 2016 [34]
			Acute treatment (20 mg/kg bw, p.o.) in male Wistar rats reduced aspirin-induced stomach ulceration and bleeding, as well as DNA damage in the stomach, liver and kidney.	Water (60 °C) extract	Hesperidin (HPLC)	The gastric effect of extract was comparable to that of 20 mg/kg bw hesperidin.	Shimamura et al., 2021 [36]
*Citrus × paradisi* Macfad (grapefruit)	Peel	Used to treat malaria [40].	In vitro DPPH, ABTS and oxygen radical scavenging activity; In vitro Fe^3+^ reducing activity; In vitro pancreatic lipase inhibition; Antioxidant activity in HepG2 cells.	70% ethanol flavonoid-rich extract	Hesperidin and hesperetin (HPLC)		Huang et al., 2020 [39]
			Treatment showed ex vivo coronary vasodilator effect and suppressed vascular resistance in isolated heart tissue; In situ, treatment decreased mean arterial pressure in mongrel dogs.	60% ethanol extract			Díaz-Juárez et al., 2009 [41]
			15-day supplementation of diet containing 8% of peel powder ameliorated TNBS-induced colitis in male Wistar rats by reducing colonic and serum expression of pro-inflammatory markers and improving colonic and serum antioxidant status.	Peel powder	Protocatechuic acid, isorhamnetin-3, kaempferol-3, feruloyl hexoside, caffeoyl hexoside, coumaroyl hexoside, myricetin-3, quercetin-3 and laricitrin-3 hexosides; quercetin-3 and isorhamnetin-3 glucuronides; quercetin-3-rutinoside; caftaric, coutaric, ferulic, syringic, coumaric and fertaric acids; epicatechin, catechin, resveratrol, procyanidin and viniferin		Maurer et al., 2020 [42]
			12-day treatment (400 mg/kg bw, p.o.) in STZ-induced diabetic male Wistar rats reduced BG and improved wound healing by promoting tissue growth and collagen synthesis.	Ethanol extracts	Carotenoids and vitamin C (non-specialized in vitro spectrometric methods)		Ahmad et al., 2013 [28]
			3-day pre-treatment (100 and 200 mg/kg bw, p.o.) of peel fraction protected against isoproterenol-induced myocardial injury in male Wistar rats by dose-dependently improving mean arterial BP, left ventricular function, cardiac biomarker enzymes and antioxidant status and myocardium histology.	Narirutin-rich fraction	Narirutin and hesperidine (HPTLC)	The myocardial protective effects of the 200 mg/kg bw fraction pre-treatment was comparable to that of 5 mg/kg bw Atenolol.	Shaikh et al., 2019 [43]
	Seed	Used in the management of diabetes and obesity [44].	30-day treatment (100, 300 and 600 mg/kg bw, p.o.) in normal male Wistar rats dose-dependently reduced FBG, BMI, AI, CRI, LDL cholesterol and total cholesterol and increased HDL cholesterol.	Methanol extract			Adeneye, 2008 [44]
			30 min in situ intragastric pre-treatment (50–500 µL) ameliorated acute hemorrhagic pancreatitis in male Wistar rats by improving pancreatic blood flow and DNA synthesis and reducing LPO.	Commercial grapefruit seed extract (Herb-Pharma s.r.o., Velke Ludnice, Slovakia)			Dembinski et al., 2004 [45]
			7-day pre-treatment (200, 400 and 600 mg/kg bw) protected against paracetamol-induced hepatotoxicity in male Wistar rats by reducing serum level of ALT, AST and ALP.	70% ethanol extract		Up to 3200 mg/kg bw of extract was non-toxic or non-lethal in mice after 72 h of oral adm.	Udom et al., 2018 [46]
*Citrus limon* (L.) Osbeck (lemon)	Peel	Used to treat hypertension [47].	In vitro DPPH, ABTS and oxygen radical scavenging activity; In vitro Fe^3+^ reducing activity; In vitro pancreatic lipase inhibition; Antioxidant activity in HepG2 cells.	70% ethanol flavonoid-rich extract	Hesperidin and hesperetin (HPLC)		Huang et al., 2020 [39]
			In vitro DPPH radical scavenging activity; Exerts in vitro immunostimulatory effect by reducing cytotoxicity and increasing proliferation of isolated mouse splenocytes.	Ethanol extracts			Diab, 2016 [48]
			In vitro inhibition of isolated rat heart ACE, PDE-5 and monoamine oxidase activities; In vitro inhibition of sodium nitroprusside- and FeSO_4_-induced LPO in isolated rat heart.	Acidified methanol (80:20% *v*/*v* of methanol and 1 N HCl solution, respectively) extract			Ademosun et al., 2019 [47]
			12-day treatment (400 mg/kg bw, p.o.) in STZ-induced diabetic male Wistar rats reduced BG and improved wound healing by promoting tissue growth and collagen synthesis.	Ethanol extracts	Carotenoids and vitamin C (non-specialized in vitro spectrometric methods)		Ahmad et al., 2013 [28]
	Seed		28-day treatment (100, 200 and 400 mg/kg bw, p.o.) of extract in STZ-induced diabetic rats reduced BG, HbA1c, intestinal α-glucosidase activity and serum TG, HDL cholesterol and enzyme biomarkers of renal and hepatic injury, increased serum insulin and improved erythrocyte, renal and hepatic antioxidant status.	Water extract		The BG-lowering and intestinal α-glucosidase inhibitory effect of extract was stronger and/or comparable to that of 20 mg/kg bw Acarbose; Up to 1000 mg/kg bw of extract was not lethal in rats after 72 h post-adm.	Demir and Celik, 2019 [49]
*Citrus × aurantiifolia* (Christm.) Swingle (Key lime)	Peel	Essential oil from peel is used for treating colds, sore throat, bronchitis, asthma and arthritis, as well as managing obesity [50]; the peel is used to treat hypertension [47].	In vitro DPPH and ABTS radical scavenging activity; 8 wks feeding of diet containing essential oil (0.74% and 2.23% per 100 g diet) ameliorated hyperlipidemia in HFD-fed male Wistar rats by increasing serum HDL cholesterol and reducing serum TG, LDL and total cholesterol and AI.	Peel essential oil	Limonene, *γ*-terpinene and *β*-pinene (GC-MS)	Cholesterol and TG was reduced to normal or near-normal range; The 2.23% per 100 g diet of oil reduced AI by 86%.	Lin et al., 2019 [51]
			4 wks oral adm. of peel powder in overweight and obese adolescents reduced BMI, systolic and diastolic BP and total and LDL cholesterol.	Peel powder			Hashemipour et al., 2016 [52]
			60-day adm. of peel powder (1 g per day, p.o.) in hypercholestorolemic male New Zealand rabbits reduced fatty streaks in the coronary arteries and aorta and improved serum antioxidant status, TG and cholesterol.	Peel powder			Boshtam et al., 2013 [53]
			In vitro DPPH radical scavenging activity.	Methanol extract	Gallic acid, 3,4-dihydroxybenzoic acid, (+)-catechin, 1,2-dihydroxybenzene, syringic acid, caffeic acid, rutin, *p*-coumaric acid, *trans*-ferulic acid, resveratrol, quercetin, kaempferol, apigenin-7-glucoside, isorhamnetin, naringenin and *trans*-cinnamic acid (HPLC-PDA)		Al Juhaimi et al., 2018 [32]
			In vitro inhibition of isolated rat heart ACE, PDE-5 and monoamine oxidase activities; In vitro inhibition of sodium nitroprusside- and FeSO_4_-induced LPO in isolated rat heart.	Acidified methanol (80:20% *v*/*v* of methanol and 1 N HCl solution, respectively) extract			Ademosun et al., 2019 [47]

AAPH, 2,2′-azobis(2-amidinopropane) dihydrochloride; ABTS, 2,2′-azinobis-(3-ethylbenzothiazoline-6-sulfonic acid); ACC, acetyl-CoA carboxylase; ACE, angiotensin converting enzyme; adm., administration; AI, atherogenic index; ALP, alkaline phosphatase; ALT, alanine transaminase; AMPK, AMP-activated protein kinase; AST, aspartate transaminase; Bcl-2, B-cell lymphoma 2; BG, blood glucose; BMI, body mass index; BP, blood pressure; BSA, bovine serum albumin; C/EBPs, CCAAT-enhancer-binding proteins; CRI, coronary risk index; DPPH, 2,2-diphenyl-1-picrylhydrazyl; FAS, fatty acid synthase; FBG, fasting blood glucose; GC-MS, gas chromatography–mass spectrometry; GLUT-4, glucose transporter type 4; GT, glucose tolerance; GSH, reduced glutathione; HbA1c, glycated hemoglobin; HDL, high-density lipoprotein; HFD, high-fat diet; HPLC-PDA, high-performance liquid chromatography-photodiode array detector; HPTLC, high-performance thin-layer chromatography; HUVECs, human umbilical vein endothelial cells; IR, insulin resistance; InRec, insulin receptor; UHPLC-QTOF-MS, ultra-high performance liquid chromatography–quadrupole time-of-flight mass spectrometry; LDL, low-density lipoprotein; LPO, lipid peroxidation; LPS, lipopolysaccharide; PPARγ, peroxisome proliferator-activated receptor γ; p.o., oral administration; ROS, reactive oxygen species; STZ, streptozotocin; TG, triglyceride; TNBS, 2,4,6-trinitrobenzenesulfonic acid.

**Table 2:** Ethnomedicinal, pharmacological and phytochemical profiles of the seeds and peel of some fruits belonging to the *Prunus* genus.

Fruit Scientific Name (Common Name)	Parts Used	Traditional Use	Pharmacological Action	Material, Extract, Fraction, Isolate or Formulation Used	Phytochemical Profile of Material, Extract, Fraction, Isolate or Formulation Used (Method of Analysis)	Remarks	References
*Prunus armeniaca* L. (apricot)	Seed/kernel	Used as an expectorant, antitussive and laxative [58].	4 wk feeding of basal diet containing 0.5–1.5 mg/kg bw of seed flour dose-dependently ameliorated dimethylnitrosamine-induced hepatic fibrosis in male SD rats by improving hepatic histology, increasing serum antioxidant enzyme activity and reducing LPO.	Seed flour			Abdel-Rahman, 2011 [59]
			8–12 doses of 60 mg/kg bw reduced blood HDL cholesterol level in adult women after 42 days.	Seed flour		HDL cholesterol reduction was significant (*p* ˂ 0.05).	Kopčeková et al., 2021 [60]
			In vitro ACE inhibitory activity.	Protein or peptide isolates			González-García et al., 2018 [61]
			Acute (1 mL p.o.) treatment protected against alcohol-induced gastric intestinal injury in Wistar rats by improving epithelial and mucosal histology, improving antioxidant status and reducing inflammation.	Seed oil			Karaboğa et al., 2018 [62]
			In vitro DPPH radical scavenging activity.	60% methanol extract of the oven-roasted seed	Gallic acid, 3,4-dihydrobenzoic acid, (+)-catechin, syringic acid, caffeic acid, rutin, *p*-coumaric acid, *trans*-ferulic acid, resveratrol, apigenin-7-glycoside, quercetin, *trans*-cinnamic acid, naringenin, kaempferol and isorhamnetin (HPLC-PDA).		Al Juhaimi et al., 2018 [63]
			In vitro scavenger of ABTS and AAPH radicals and methylglyoxal; In vitro inhibition of methylglyoxal-induced BSA glycation and AGE production.	Water extract	*p*-Hydroxybenzoic, syringic acid, vanillic acid, *p*-coumaric, caffeic acid, ferulic acid, protocatechuic acid, gallic acid and sinapinic acid (HPLC).		Mesías et al., 2013 [64]
			40 days of feeding a basal diet containing seed flour ameliorated cholesterol-induced hypercholesterolemia in male Wistar rats by reducing serum HDL, VLDL and total cholesterols and TG and increasing HDL cholesterol.	Raw and detoxified (using 25% NaCl solution) seed flour	L-ascorbic acid and *β*-carotene (non-specialized in vitro spectrometric methods).	Raw and detoxified seeds reduced AI by 5- and 8-fold, respectively.	Tanwar et al., 2018 [65]
			Acute (after 8 h) and 8-day i.p. treatment (2, 3 and 4 mg/kg bw) reduced BG, HbA1c and LPO and increased serum insulin and catalase activity in alloxan-induced diabetic male Swiss mice.	Detoxified (infrared-assisted) water extract	Chlorogenic acid, amygdaline and procyanidin derivatives (HPLC-PDA).	The antidiabetic and antioxidant effects of the extracts were comparable and/or more potent than those of 5 mg/kg bw Glibenclamide	Raafat et al., 2018 [58]
			In vitro ABTS radical scavenging and Fe^3+^ reducing antioxidant activity; In vitro ACE inhibitory activity; In vitro hypocholesterolemic activity.	Protein hydrolysates			García et al., 2016 [66]
*Prunus cerasus* L. (sour cherry)	Seed	Used to prepare syrup and herbal tea infusion for treating fever, liver disease and gonorrhea [67].	5 wk supplementation with seed powder (0.1 mg/g diet) ameliorated HFD-induced obesogenic and neuroinflammatory effects in male Wistar rats by reducing serum cholesterols and TG, systolic BP and endothelial inflammatory makers (VCAM-1 and ICAM-1) of the frontal cortex and hippocampus.	Seed powder			Micioni Di Bonaventura et al., 2020 [68]
			17 wk feeding of basal diet containing seed flour attenuated HDF-induced adipogenesis in rats by downregulating the expression of adipogenic genes in adipose tissue.	Seed flour			Cocci et al., 2021 [69]
			Oral adm. (200 mg/kg bw) of seed extract ameliorated HCl and ethanol-induced gastric lesions in Swiss albino male mice. Also reduced carrageenan-induced nociceptive pain, inflammation and oxidative stress by reducing pro-inflammatory makers (TNF-α and IL-6) and improving antioxidant status.	Ethyl acetate extract	Oleic acid, linoleic acid, linolenic acid, stearic acid and palmitic acid (GC-FID).	The gastroprotective and anti-nociceptive effects of the extract were comparable to those of 50 mg/kg bw Ranitidine and 100 mg/kg bw Ibuprofen, respectively.	Raafat et al., 2020 [70]
			50-day treatment (30 mg/kg bw p.o.) protected against ischemic diabetic retinopathy in Zucker diabetic fatty male rats by improving GT, increasing HO-1 and reducing retinal thickness.	Defatted flavonoid-rich seed extract			Varga et al., 2017 [71]
			In vitro ACE inhibitory activity.	Protein or peptide isolates			González-García et al., 2018 [61]
			Acute and 8-day adm. (100, 150 and 200 mg/kg bw, i.p.) reduced BG levels and improved antioxidant status and pancreatic histology in alloxan-induced diabetic mice.	Ethyl acetate extract		The 200 mg/kg bw extract significantly (*p* ˂ 0.05) outperformed 5 mg/kg bw Glibenclamide in BG lowering.	Saleh et al., 2017 [72]
			In vitro scavenger of ABTS and AAPH radicals and methylglyoxal; In vitro inhibition of methylglyoxal-induced BSA glycation and AGE production.	Water extract	*p*-Hydroxybenzoic, syringic acid, vanillic acid, *p*-coumaric, caffeic acid, ferulic acid, protocatechuic acid, gallic acid, sinapinic acid and gentisic acid (HPLC).		Mesías et al., 2013 [64]
			Reduced lipopolysaccharide-induced inflammatory response in leukocytes isolated from diabetic patients by inhibiting upregulation of pro-inflammatory biomarkers (TNF-α and IL-8) and increasing HO-1 expression.	Defatted flavonoid-rich seed extract (Hydromethanolic)			Mahmoud et al., 2012 [73]
			16 wks of feeding a basal diet containing 30 mg/kg seed extract afforded cardioprotective effects in cholesterol-fed male rabbits by improving cardiac function, increasing HO-1 expression and reducing cardiac atherosclerotic plaque formation.	Defatted flavonoid-rich seed extract			Juhasz et al., 2013 [74]
*Prunus persica* (L.) Batsch 1801 (peach)	Seed	Used to treat amenorrhea and rheumatoid arthritis [70].	In vitro ACE inhibitory activity.	Protein or peptide isolates			González-García et al., 2018 [61]
			In vitro hydroxyl and ABTS radical scavenging activity; In vitro Fe^3+^ reducing activity; In vitro inhibition of linoleic acid lipid peroxidation; Inhibition of ROS production in HeLa cells.	Protein or peptide isolates			Hernández-Corroto et al., 2018 [75]
			In vitro scavenger of ABTS and AAPH radicals and methylglyoxal; In vitro inhibition of methylglyoxal-induced BSA glycation and AGE production.	Water extract	*p*-Hydroxybenzoic, syringic acid, vanillic acid, *p*-coumaric, caffeic acid, ferulic acid, protocatechuic acid, gallic acid and sinapinic acid (HPLC).		Mesías et al., 2013 [64]
			In vitro ACE inhibitory activity.	Protein or peptide isolates			Vásquez-Villanueva et al., 2019 [76]
			In vitro ABTS radical scavenging and Fe^3+^ reducing antioxidant activity; In vitro ACE-I inhibitory activity; In vitro hypocholesterolemic activity.	Protein hydrolysates			García et al., 2016 [66]
			In vitro DPPH and ABTS radical and scavenging activity; In vitro Fe^3+^ reducing and Fe^2+^ chelating activity.	Ethanol extract			Loizzo et al., 2015 [77]
			Radical scavenging and antioxidant activity in isolated rat liver cells with CCl_4_-induced oxidative stress; Sub-chronic adm. (400 mg/kg bw, p.o.) of extract ameliorated CCl4-induced hepatic damage in SD rats by increasing the expression Nrf2 and NQO1 and improving hepatic histology.	Hydromethanolic extract		Up to 4000 mg/kg bw (p.o.) acute adm. of extract was not toxic or caused mortality in rats; Cellular antioxidant activity of extract was comparable to that of gallic acid.	Rehman et al., 2021 [78]
			Inhibited histamine release and the expression of pro-inflammatory cytokines (TNF-α and IL-6) in human mast cells.	Methanol extract and isolated phenolic glycosides (vanilloloside and lacticolorin)	Vanilloloside and lacticolorin (HPLC, NMR).		Kim et al., 2013 [79]
*Prunus domestica* L. (European plum)	Seed or pit		Fe^3+^ reducing antioxidant property; Oxygen and DPPH radicals scavenging activity; Xanthine oxidase inhibitory activity.	Methanol extract of defatted sample	*p*-Hydroxybenzoic acid, vanillin, vanillic acid, 3,4-dihydroxybenzoic acid, gallic acid and syringic acid (HPLC-ESI-MS).		Khallouki et al., 2012 [80]
			In vitro ACE inhibitory activity.	Protein or peptide isolates			González-García et al., 2018 [61]
			In vitro ABTS radical scavenging and Fe^3+^ reducing antioxidant activity; In vitro ACE-I inhibitory activity; In vitro hypocholesterolemic activity.	Protein hydrolysates			García et al., 2016 [66]

AAPH, 2,2′-azobis(2-amidinopropane) dihydrochloride; ABTS, 2,2′-azinobis-(3-ethylbenzothiazoline-6-sulfonic acid); ACE, angiotensin converting enzyme; adm., administration; AGEs, advanced glycation end-products; AI, atherogenic index; BG, blood glucose; BP, blood pressure; BSA, bovine serum albumin; DPPH, 2,2-diphenyl-1-picrylhydrazyl; GC-FID, gas chromatography–flame ionization detection; GT, glucose tolerance; HbA1c, glycated hemoglobin; HDL, high-density lipoprotein; HFD, high-fat diet; HO-1, heme oxygenase; HPLC-PDA, high-performance liquid chromatography–diode array detection; HPLC-ESI-MS, HPLC–electrospray ionization–mass spectrometry; VLDL, very-low-density lipoprotein; LPO, lipid peroxidation; NMR, nuclear magnetic resonance; p.o., oral administration; ROS, reactive oxygen species; SD, Sprague Dawley; TG, triglyceride.

## Data Availability

The data in this study are available from the corresponding author on request.

## Abbreviations

ACE, angiotensin converting enzyme; AGEs, advanced glycation end-products; AI, atherogenic index; BG, blood glucose; BMI, body mass index; BP, blood pressure; CRI, coronary risk index; FBG, fasting blood glucose; HFD, high-fat diet; LPO, lipid peroxidation; LPS, lipopolysaccharide; ROS, reactive oxygen species; SD, Sprague Dawley; SHR, spontaneously hypertensive rats; STZ, streptozotocin; TG, triglyceride.
